# Cellular and Molecular Effects of the Bruck Syndrome-Associated Mutation in the *PLOD2* Gene

**DOI:** 10.3390/ijms252413379

**Published:** 2024-12-13

**Authors:** Olga I. Bolshakova, Evgenia M. Latypova, Artem E. Komissarov, Alexandra D. Slobodina, Elena V. Ryabova, Elena Yu. Varfolomeeva, Olga E. Agranovich, Sergey F. Batkin, Svetlana V. Sarantseva

**Affiliations:** 1 Petersburg Nuclear Physics Institute Named by B.P. Konstantinov of National Research Centre «Kurchatov Institute», 188300 Gatchina, Russia; bolshakova_oi@pnpi.nrcki.ru (O.I.B.); latypova_em@pnpi.nrcki.ru (E.M.L.); komissarov_ae@pnpi.nrcki.ru (A.E.K.); sashylikslobodina@mail.ru (A.D.S.); ryabova_ev@pnpi.nrcki.ru (E.V.R.); varfolomeeva_ey@pnpi.nrcki.ru (E.Y.V.); 2 Turner National Medical Research Center for Children’s Orthopedics and Trauma Surgery, 196603 Saint Petersurg, Russia; olga_agranovich@yahoo.com (O.E.A.); sergey-batkin@mail.ru (S.F.B.)

**Keywords:** Bruck syndrome, mutations, PLOD2, transfection, cells

## Abstract

Bruck syndrome is a rare autosomal recessive disorder characterized by increased bone fragility and joint contractures similar to those in arthrogryposis and is known to be associated with mutations in the *FKBP10* (*FKBP prolyl isomerase 10*) and *PLOD2* (*Procollagen-Lysine,2-Oxoglutarate 5-Dioxygenase 2*) genes. These genes encode endoplasmic reticulum proteins that play an important role in the biosynthesis of type I collagen, which in turn affects the structure and strength of connective tissues and bones in the body. Mutations are associated with disturbances in both the primary collagen chain and its post-translational formation, but the mechanism by which mutations lead to Bruck syndrome phenotypes has not been determined, not only because of the small number of patients who come to the attention of researchers but also because of the lack of disease models. In our work, we investigated the cellular effects of two forms of the wild-type *PLOD2* gene, as well as the *PLOD2* gene with homozygous mutation c.1885A>G (p.Thr629Ala). The synthesized genetic constructs were transfected into HEK293 cell line and human skin fibroblasts (DF2 line). The localization of PLOD2 protein in cells and the effects caused by the expression of different isoforms—long, short, and long with mutation—were analyzed. In addition, the results of the transcriptome analysis of a patient with Bruck syndrome, in whom this mutation was detected, are presented.

## 1. Introduction

Bruck syndrome (BRKS) is a poorly understood autosomal recessive disorder that combines features of osteogenesis imperfecta and arthrogryposis multiplex congenita (AMC) [1,2]. BRKS can be considered, among other things, as OI type XI (OMIM # 610968) [2,3]. The disease is characterized by bone fragility leading to multiple fractures, particularly of the ribs and long (tubular) bones, which appear as early as infancy or early childhood. As a rule, patients have multiple joint contractures and cutaneous pterygia, the same as in AMC [2,4], among which those mostly observed are flexion (contracture) deformities of the large joints (knee, elbow, and ankle joints) and, to a lesser extent, flexion (contracture) deformities of the small joints of the hand (camptodactyly, adduction contracture of the first finger) [5]. Most registered patients do not have intellectual disabilities or reduced height at birth [2,6]. However, some have vertebral malformations (platyspondyly), leading to postnatal short stature, dwarfism, and progressive kyphoscoliosis [5].

Some patients have blue sclera similar to those observed in osteogenesis imperfecta. BRKS differs from osteogenesis imperfecta in that patients do not have hearing loss or dentinogenesis imperfecta, and in the presence of clubfoot [6]. Treatment of patients with BRKS includes physical therapy, orthotics, surgical treatment (corrective osteotomies), and the use of bisphosphonates and drugs containing vitamin D and calcium [7].

BRKS is divided into types I and II, which develop due to mutations in the *FKBP10* and *PLOD2* genes, respectively. The classification of BRKS is based only on genetic data, since clinical data do not allow for a phenotypic distinction between patients with these two types [5,8,9,10]. At the same time, different mutations in these two genes lead to a wide range of BRKS phenotypes, and there is variability in the degree of bone mineral density loss, fracture frequency, presence of pterygium, and disease severity. There is a heterogeneity in patient phenotypes even within the same family [6,11,12].

The *FKBP10* and *PLOD2* genes are located on chromosomes 17q21.2 and 3q24, respectively, and encode endoplasmic reticulum resident proteins that play an important role in the biosynthesis of type I collagen [5,13]. The *PLOD2* gene encodes lysyl hydroxylase-2 (LH2), which is an enzyme responsible for the hydroxylation of lysine residues in type I collagen telopeptides [4,14]. The resulting hydroxylysine residues perform two important functions in collagen: they participate in the formation of intermolecular collagen cross-links, and their hydroxyl groups serve as sites of attachment for carbohydrate units [9,15]. Thus, mutations in *PLOD2* can alter the hydroxylation level of type I collagen and lead to the abnormal cross-linking of collagen fibrils, which increases bone fragility and the risk of fractures [4,5,11].

Different splicing variants of the *PLOD2* gene result in two forms of the protein: LH2a and LH2b. The longer form, LH2b, contains 758 amino acids, of which 21 amino acids are encoded by the additional exon 13A. The shorter form, LH2a (737 amino acids), lacks this additional region, similar to other lysyl hydroxylases (LH1 and LH3) [9,10,15,16]. LH2b is considered ubiquitously expressed, while LH2a mRNA has been detected in the kidney, spleen, liver, cartilage, and placenta, similar to LH2b, but is absent from the skin, lung, aorta, and dura mater [4,6,9,16,17].

Most mutations in the *PLOD2* gene are present in a homozygous state, while mutations in a compound heterozygous state have been described. Most mutations are missense mutations and are concentrated mainly in exons 17–19, encoding the C-terminal domain with LH activity. However, nonsense mutations, splice site mutations, deletions, and duplications also occur in other regions of the *PLOD2* gene [3,13]. At the same time, no obvious phenotypic differences were found between patients with mutations affecting only the long isoform of LH2 and patients with mutations affecting both isoforms of LH2 [4,11]. It should be noted that the precise functions of the domains in LH2 are not completely clear; therefore, the mechanisms by which mutations in PLOD2 lead to BRKS are still not fully understood, nor is the relationship between defective collagen cross-linking and contractures, the presence of which was long considered a characteristic feature of this disease. However, in recent years, patients with a BRKS phenotype without congenital contractures have been identified [3,10].

Functional studies of mutations leading to BRKS are associated with objective difficulties, since the number of patients with BRKS is limited, and the clinical phenotype of the patient is not always clear [8].

Studies of the effects of mutations in the *PLOD2* gene on cell cultures are rare; therefore, in this study, we created an in vitro model of BRKS with a mutation c.1885A>G (p.Thr629Ala) in the *PLOD2* gene to characterize the cellular effects of this mutation [12]. We also performed transcriptome analysis of a muscle sample from a BRKS patient with this mutation, identifying the key molecular processes and pathways affected by *PLOD2* disruption.

## 2. Results and Discussion

### 2.1. In Vitro BRKS Model

**Figure 1:**
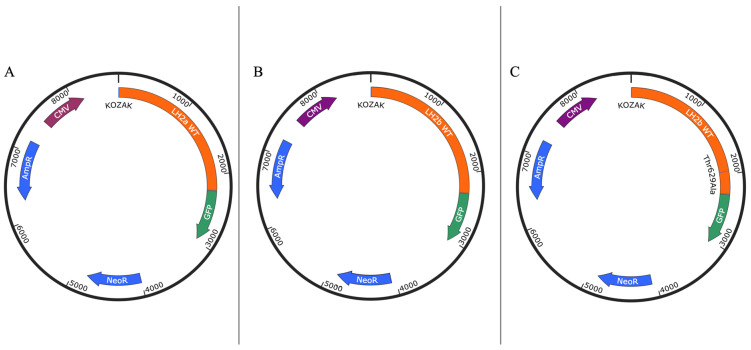
Schematic representation of genetic constructs CMV—CMV promoter: (**A**) wt *PLOD2* (*LH2a*) expression plasmid, (**B**) wt *PLOD2* (*LH2b*) expression plasmid, and (**C**) mutant Thr629Ala allele *PLOD2* expression plasmid.

In the first stage of this work, we obtained genetic constructs encoding the native and mutant variants of the human *PLOD2* gene fused in-frame to the *GFP* gene (Figure 1).

The genetic constructs were then transfected into HEK293 and DF2 cell lines. Of greatest interest to us was modeling the effects of the *PLOD2* gene on fibroblasts. Fibroblasts are the main collagen-producing cells in the skin and are responsible for the production of extracellular matrix in connective tissue by secreting collagen [18]. Importantly, DF2 is a diploid, not a continuous, human line. In addition, it has been previously shown that not only are mutations in the *PLOD2* gene associated with bone collagen impairment in BRKS, but *PLOD2* also plays a significant role in fibrotic processes in systemic scleroderma [14].

**Figure 2:**
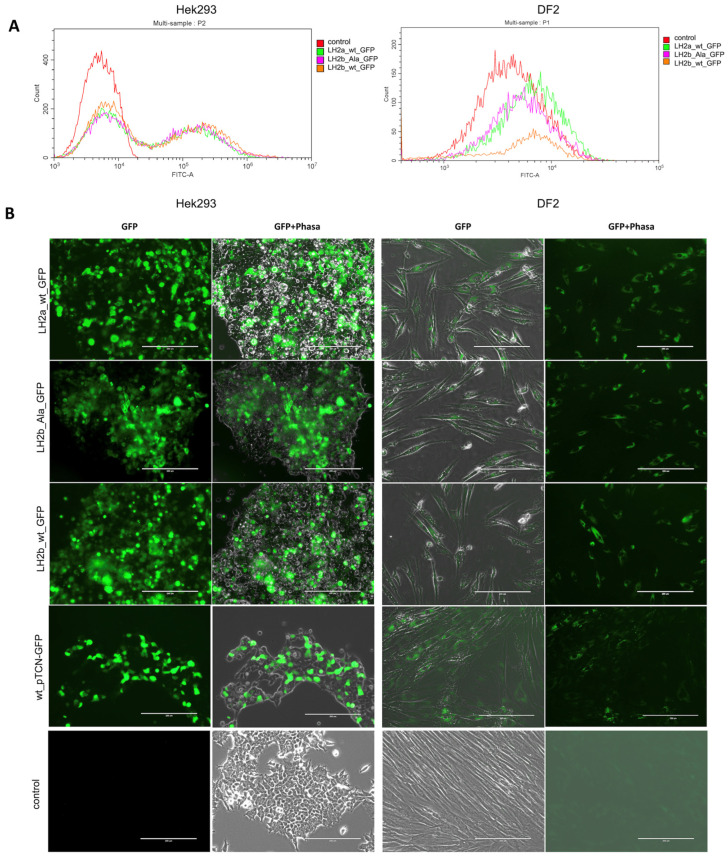
Transfection efficiency of a plasmid with a *GFP* reporter. Flow cytometry analysis of transfection efficacy of HEK293 (**left**) and DF2 (**right**) cells in 24 h (**A**). Visualization of HEK293 (**left**) and DF2 (**right**) cells in 48 h using the EVOS FL Auto Imaging System (**B**). Green – PLOD2 fused to GFP. Scale bar—200 µm.

The efficiency of DF2 cell transfection with all three genetic constructs was about 100% (Figure 2A), while protein expression in them was low (Figure 2B), which, along with the high autofluorescence of these cells, led to difficulties in visualizing some effects. Cells were additionally stained with antibodies to the PLOD2 protein to confirm transfection (Figure S1).

The HEK293 line is the most commonly used for transfection and yields positive results, so it was chosen as one of the variants of our experiment. According to the flow cytometry data, the transfection efficiency of these cells after 24 h was approximately 50% for all three constructs (Figure 2A). After another day, the number of cells with a green signal in all variants was significantly higher (Figure 2B). Subsequently, the cells were kept on the selective medium, which increased the reliability of the assays. Most analyses were carried out in fluorescence mode and, consequently, the results obtained relate to transformed cells.

In all cases, we worked with transient transfections, since we failed to create a stable transfected HEK293 line, and this was not the task for the DF2 line.

### 2.2. Analysis of the Localization of the PLOD2 Protein

**Figure 3:**
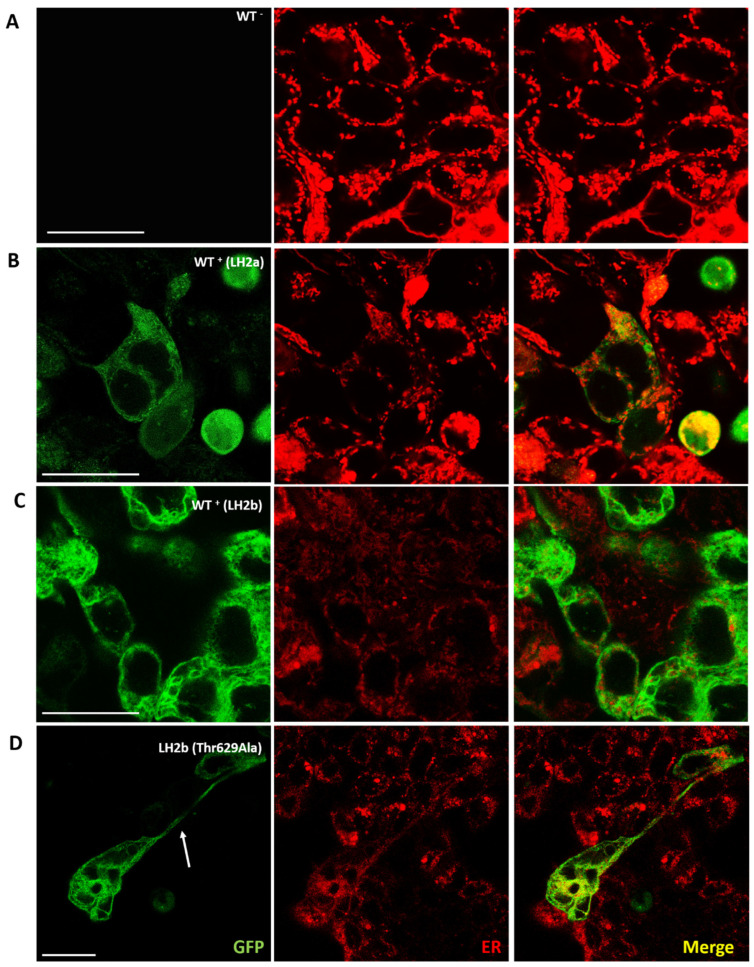
Confocal microscopy images of HEK293 cells transfected with three different constructs, including GFP-LH2a (**B**), GFP-LH2b (**C**), and GFP-LH2b with mutation Thr629Ala (**D**) and wild-type without constructs (**A**). In Figure (**D**), the arrow shows a change in the morphology of the cell with expression of the LH2b (Thr629Ala). The left column (green) shows the images of GFP-tagged protein PLOD2, the middle column (red) shows the images of ER, and the right column shows the overlaid images of the left and middle columns. Scale bar—25 µm.

Since the protein encoded by the *PLOD2* gene is necessary for the biogenesis of normal mature collagen that forms the extracellular matrix, as well as for the stability of bonds within collagen fibers, mutations in it can be involved in various pathological processes, primarily in changing its localization in the cell and disrupting its transport into the extracellular matrix. It was previously shown that the wild-type *PLOD2* gene product is found in the cell cytoplasm [8]. We also observed both wild-type protein variants and a mutated form in the cell cytoplasm. PLOD2 is an ER-resident protein localized, according to various sources, to the cisternae or membranes of the rough ER. We analyzed the localization of PLOD2 in the ER. We hypothesized that in our experiments, the localization of this protein within the cell, namely, its extraction into the extracellular matrix with the formation of large vacuolar structures adjacent to the ER, might be disrupted, particularly as a result of mutation. However, we found no change in the localization of PLOD2. An analysis of PLOD2 variant localization in HEK293 cells is shown in Figure 3. The images show that the protein occupies the entire volume of the ER, repeating its pattern. We did not find any difference in the distribution between the protein isoforms, nor did we observe any clear disruption of the ER structure, but it was noted that in transfected cells, it was stained significantly weaker. The reasons for this phenomenon require additional research.

**Figure 4:**
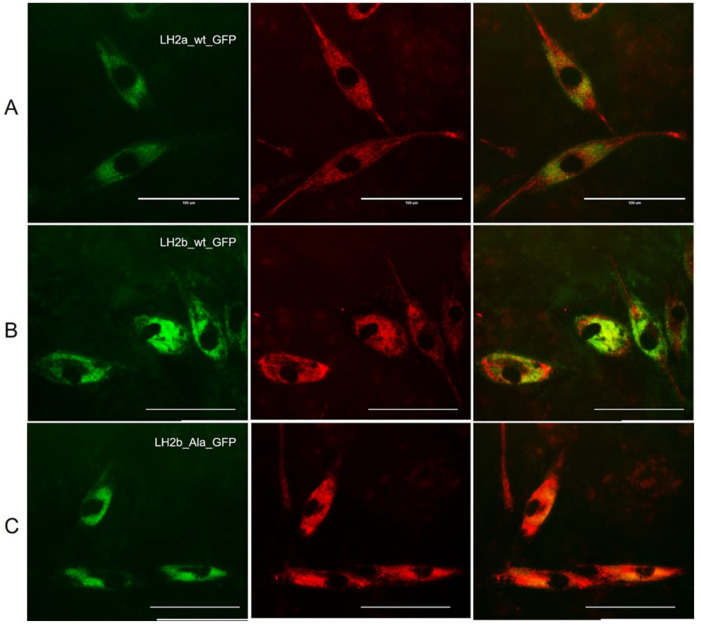
Visualization of DF2 cells transfected with three different constructs, including *GFP-LH2a* (**A**), *GFP-LH2b* (**B**), and *GFP-LH2b* with mutation Thr629Ala (**C**) using the EVOS FL Auto Imaging System. The middle column (red) shows the images of ER, and the right column shows the overlaid images of the left and middle columns. Scale bar—100 µm.

A similar pattern of protein localization was observed in DF2 cells (Figure 4). It should be noted that ER staining was stronger in fibroblasts.

### 2.3. Analysis of Cellular Effects of the PLOD2 Gene

#### 2.3.1. Analysis of Cell Viability After Transfection

**Figure 5:**
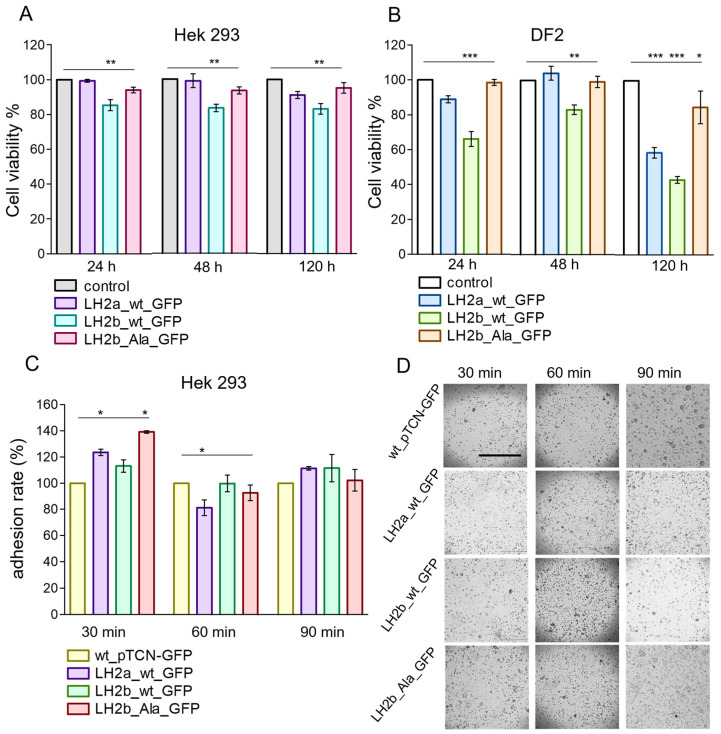
Cell viability assays of HEK293 (**A**) and DF2 (**B**) 24, 48, and 120 h after transfection. Cell adhesion rate in each group of the HEK293 cells at 30, 60, and 90 min after transfection (**C**,**D**). Scale bar—1000 µm; mean ± SEM, Student’s *t*-test. * *p* ≤ 0.05, ** *p* ≤ 0.01, *** *p* ≤ 0.001. N = 6 (**A**,**B**) and N = 12 (**C**).

Cell viability was determined by the MTT colorimetric method, which evaluates their metabolic activity. In HEK293 cells, we observed a decrease in viability only for cells modified with the long form of the wild-type protein (LH2b_wt_GFP; Figure 5A). In DF2 cells, 24 h after transfection, the metabolic activity depended on the inserted genetic construct (Figure 5A). For LH2b_Ala_GFP, viability remained at the control level, for LH2a_wt_GFP, it decreased slightly, and for LH2b_wt_GFP, it was reduced by more than 30% (Figure 5A). After 48 h, some increase in metabolic activity was observed in cells expressing wild-type forms of the protein, and for the form with the mutation, it remained at the same level as after 24 h, i.e., at the control level. At 120 h after transfection, the metabolic activity of cells with the LH2b_Ala_GFP construct remained normal, and for the wild-type one, it significantly decreased. These effects were reflected in the morphology of the cells and their growth patterns. The transfected cells increased in size and did not form a dense monolayer.

The GFP signal of cells expressing the mutant PLOD2 variant was weaker in comparison with that of the wild-type protein. This indirectly confirms the data obtained by Wang et al. [8], who described a decrease in the expression of the PLOD2 Arg619His protein compared to the wild-type protein.

We hypothesized that the decrease in cell viability in DF2 and HEK293 lines is associated with gene overexpression, and the deterioration in monolayer formation may be the result of the disruption of intercellular interactions and the changes in their adhesive properties and ability to migrate.

#### 2.3.2. Migration and Adhesion Analysis

Both PLOD2 and FKBP10 are thought to indirectly interact with molecules associated with cell proliferation, migration, invasion, and adhesion, although the exact mechanism of these interactions is unclear. PLOD2 has been shown to increase invasion and migration in several cancer cell lines [19,20] and promote tumor metastasis by enhancing cell migration directly and indirectly by inducing collagen reorganization [21,22]. It is known that patients with BRKS need to be closely monitored due to the frequent occurrence of skin lesions.

Cell–cell adhesion is a critical process, the disruption of which can cause diseases such as cancer, arthritis, and osteoporosis. It participates in fundamental processes in the cell and is necessary for proliferation, migration, and differentiation, while providing communication between the cell and the extracellular matrix. Therefore, it is important to determine whether the mutation in the *PLOD2* gene affects this process. We analyzed the adhesion rate on HEK293 cells after transfection with genetic constructs containing *PLOD2*, compared with those containing only *GFP*. The results are shown in Figure 5C,D. In the first 30 min, we observed an increase in the adhesion rate in transfected cells. For LH2a_wt_GFP and LH2b_Ala_GFP, the increase was reliable (*p* less than 0.05). After 60 min, the adhesion rate was reduced in cells with LH2a_wt_GFP. After 90 min, cell adhesion to the PLOD2-containing constructs did not differ from the adhesion of the comparison line. The data obtained contained some contradictions compared to the growth processes of cultures after transfection that we observed and described above. However, it should be considered that adhesion is not only the process of cell attachment to the substrate but, above all, the process of interaction between cells, and we see that transfected cells formed large and strong conglomerates during cultivation. At the same time, there is evidence that cells differed in their adhesive abilities in relation to various surfaces and substrates [23]. Perhaps our cultures reduced their adhesive properties specifically in relation to the culture plastic.

**Figure 6:**
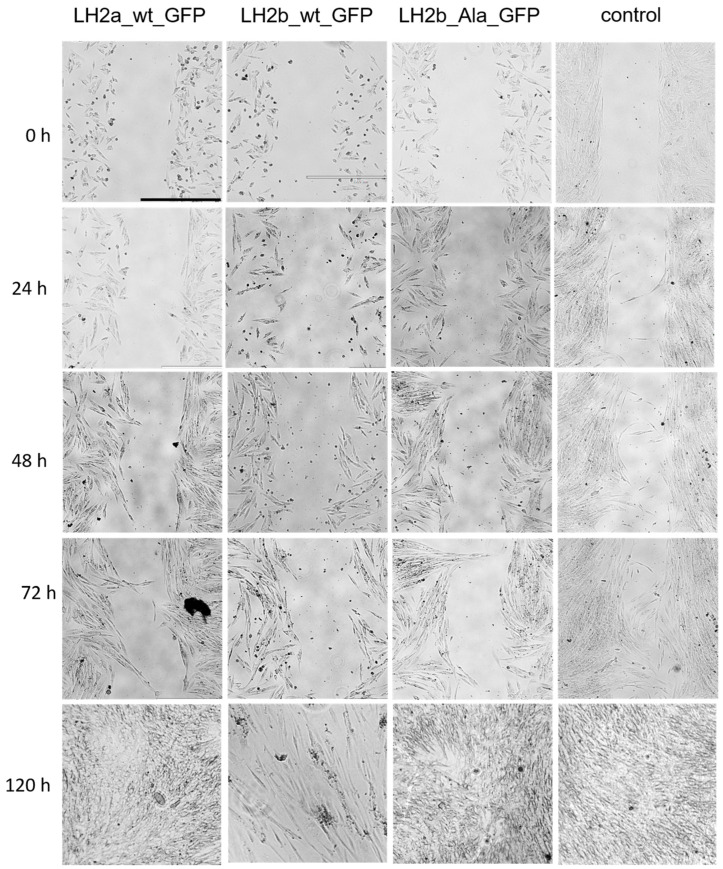
Cell migration assay of DF2. Scale bar—1000 µm. N = 4.

**Figure 7:**
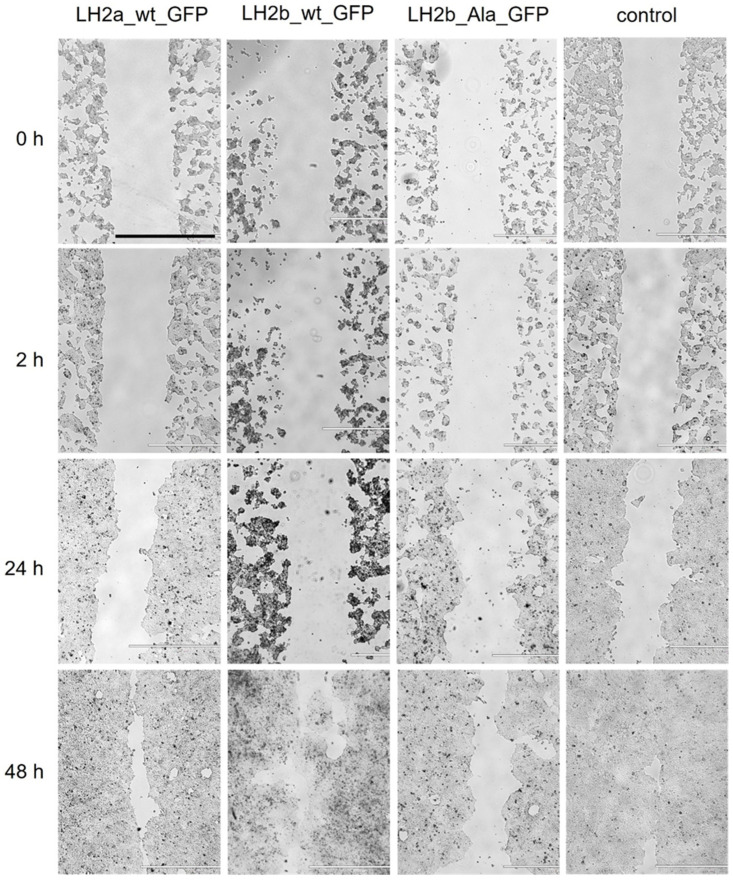
Cell migration assay of HEK293. Scale bar—1000 µm. N = 6.

The adhesion process is closely related to migration. Figure 6 and Figure 7 show the data of cell migration experiments performed by the scratch healing method for DF2 and HEK293 cell lines, respectively. Figure 6 shows that scratch healing in transfected DF2 cells was slower than in the control. After 96 h, scratches in the control cells were completely healed, while in the experimental samples, complete healing was observed only after 120 h. It could be assumed that in all cells with *PLOD2* expression, the migration process is impaired, and most significantly in cells transfected with the LH2b_wt_GFP construct. However, it should be considered that, in these cells, we observed a decrease in viability, which may affect the obtained result. At the same time, in cells with a mutation, the decrease in viability was significantly lower, and scratch healing was slower. That is, in this case, we can say that in fibroblasts, the mutated form of the protein caused a violation of cell migration.

We tested this hypothesis on HEK293 cells, which are characterized by greater viability after transfection. In this case, the migration of cells expressing the mutant protein was also reduced (Figure 7). A similar result of a decrease in cell migration in the absence of a decrease in the adhesion rate was observed by Chet et al. [24], where the overexpression of tyrosine kinase protein 7 (DOK7) led to an increase in cell adhesion to Matrigel and, at the same time, a decrease in migration was noted. Morath et al. [25] observed a decrease in the migration ability of fibroblasts when exposed to mycophenolic acid. At the same time, cell adhesion increased. Cell migration is a complex process, where adhesion is an important but not the only factor necessary for its implementation. Cell polarity, mechanical stress, and interaction with the extracellular matrix (ECM) are essential [26,27]. The state of the actin cytoskeleton of cells plays a significant role [28] due to the coordinated operation of signaling pathways and other factors. For example, polysialic acid was shown to enhance cell migration regardless of adhesion ability [29]. Bokhobza et al. used the CRISPR/Cas9 method to delete the ORAI1 gene in HEK293 cells, which regulates processes such as proliferation, cell adhesion, and migration, but this only affected cell migration and did not lead to changes in proliferation and adhesion [30]. Impaired cell migration with the mutation in *PLOD2* observed in our study may be associated with many factors and require additional study. Currently, the relationship between PLOD2 and migration has been identified mainly in tumor cells. It was shown that *PLOD2* knockdown blocks migration in NCI-H1975 cells, while overexpression increases it in A549 cells [21]. A high expression of PLOD2 in oropharyngeal squamous cell carcinoma (SCC) led to increased cell motility and promoted metastasis in vivo [31]. In Kreße et al.’s study [32], PLOD2 promoted the invasion and proliferation of tumor cells in vitro, regardless of their attachment to the substrate. PLOD2 overexpression is a marker of some cancers and is associated with their poor prognosis [33,34,35,36,37]. In our experiments performed on normal DF2 cells and normal-origin HEK293 cells, no increase in migration was detected with *PLOD2* overexpression.

Pathogenic mutations leading to BRKS have been described in a number of articles [12,14]. The mutation we studied resulted in an A/G substitution at position 1885 of the *PLOD2* gene [12] and a substitution of the Thr amino acid at position 629 of the protein (LH2b) to Ala. We tested whether Thr at position 629 of lysyl hydroxylase LH2 was conserved in the sequences of other human lysyl hydroxylases (LH1 and LH3), as well as in the sequences of lysyl hydroxylases from other organisms (from Homo sapiens *to Caenorhabditis elegans*; Table 1), and found that this amino acid is located in a highly conserved region in various organisms, which implies its importance for the functioning of lysyl hydroxylases in general and LH2 in particular.

### 2.4. Transcriptomic Analysis of a Muscle Sample from a Patient with BRKS

To better understand the molecular processes in BRKS, we performed transcriptome analysis of a muscle sample from an eight-year-old female patient with a homozygous mutation c.1885A>G (p.Thr629Ala) in the *PLOD2* gene. Genetic analysis and a detailed clinical description of this case were presented previously [12]. Samples from open databases (accession number GSE201255) of quadriceps femoris tissue from four female patients with an average age of eight years were used as a control [38].

**Figure 8:**
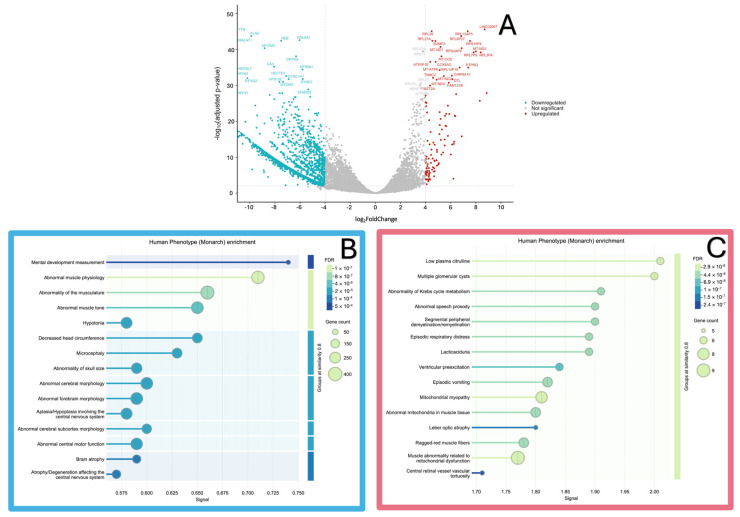
(**A**) Volcano plot showing −log10 of adjusted *p*-value vs. log2FoldChange. Blue dots represent downregulated differentially expressed genes (DEGs) and red dots represent upregulated DEGs. Enrichment analysis of human phenotype of (**B**) downregulated DEGs and (**C**) upregulated DEGs.

As a result of transcriptome analysis, it was determined that in the experimental sample, relative to the level of control samples, the expression level was statistically significantly (*p*-value ≤ 0.01 and log2FoldChange ≥ 4) increased in 112 genes and decreased (*p*-value ≤ 0.01 and log2FoldChange ≤ −4) in 2500 genes (Figure 8A, Supplementary Material 1). 

We performed an enrichment analysis for human phenotypic manifestations and noted that the terms mental development measurement, abnormal muscle physiology, abnormality of the musculature, and abnormal central motor function were found for the down-expression group, while the terms low plasma citrulline, multiple glomerular cysts, mitochondrial myopathy, and muscle abnormality related to mitochondrial dysfunction were found for the up-expression group (Figure 8B,C).

Then, for each group of genes, Gene Ontology enrichment analyses were performed in three categories: biological processes (BPs), molecular functions (MFs), and cellular components (CCs).

**Figure 9:**
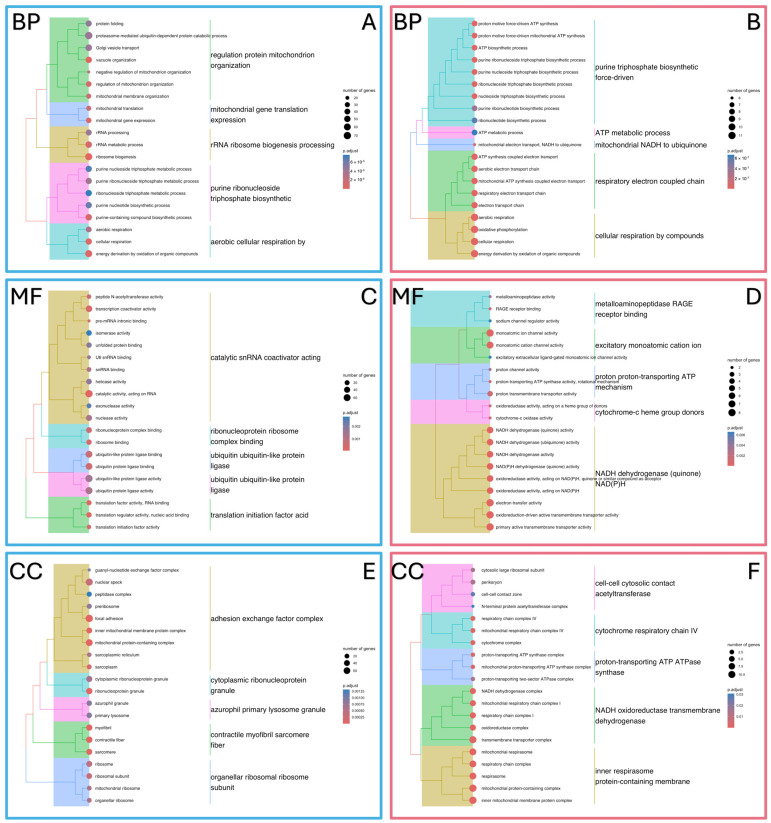
GO function and pathway enrichment analysis of down- and up-regulated DEGs. (**A**) BP of down- and (**B**) up-regulated DEGs, (**C**) MF of down- and (**D**) up-regulated DEGs, and (**E**) CC of down- and (**F**) up-regulated DEGs.

The following biological processes were identified for genes with down-expression: the proteasome-mediated ubiquitin-dependent protein catabolic process, ribosome biogenesis, cellular respiration, and energy derivation by oxidation of organic compounds (Figure 9A). In turn, the group of genes with up-expression affected the following group of processes: the nucleoside triphosphate metabolic process, mitochondrial NADH to ubiquinone, respiratory electron-coupled chain, and cellular respiration by compounds according to GO analysis (Figure 9B).

Enrichment analysis of the molecular function category for the downregulated group showed that ATP hydrolysis activity, transcription coactivator activity, and ubiquitin protein ligase activity may be involved in this disease (Figure 9C), while the upregulated group of genes affected functions such as the primary active transmembrane transporter, electron transfer activity, oxidoreductase activity acting on NAD(P)H, and proton transmembrane transporter activity (Figure 9D).

The most interesting cellular components associated with genes from the group with down-expression were as follows: focal adhesion, mitochondrial protein-containing complexes, myofibrils, contractile fibers, and sarcomeres (Figure 9E). In turn, the following components were identified for up-expression: mitochondrial protein-containing complex, transmembrane transporter complex, synaptic vesicle, and oxidoreductase complex (Figure 9F).

According to the Gene Ontology analysis, a group of 55 genes was identified that may play a significant role in the intrinsic apoptotic signaling pathway (GO:0097193, *p*.adjust = 0.000459). This pathway represents one of the mechanisms by which cells initiate programmed cell death, known as apoptosis. It is noteworthy that Figure 9 demonstrates the frequent occurrence of terms related to both up- and down-regulated gene groups, which affect mitochondrial metabolism. This observation suggests that alterations in *PLOD2* gene expression may lead to metabolic stress, subsequently initiating the apoptotic signaling pathway. This process may influence mitochondrial membrane permeability, potentially contributing to the development of oxidative stress and apoptosis.

The results of transcriptome analysis showed that DEGs affect cellular processes and components that can disrupt the normal state of mitochondria (cellular respiration, respiratory electron-coupled chain, electron transfer activity, NAD(P)H-acting oxidoreductase activity, and mitochondrial protein-containing complexes). Studies using a mouse model of osteogenesis imperfecta have shown that misfolded procollagen in osteoblasts of this model triggers an integrated stress response. Regulation occurs in the endoplasmic reticulum through mitochondrial HSP70 (mt-HSP70) and ATF5, which may suggest that mitochondria can initiate an integrated stress response in case of disruption of connections between the endoplasmic reticulum and mitochondria [39].

Interestingly, four of the five genes whose expression was most altered are associated with the functioning of muscle tissue. For example, the *MYH2* (Myosin Heavy Chain 2; log2FoldChange = −15) and *MYH1* (Myosin Heavy Chain 1; log2FoldChange = −15) genes encode the myosin heavy chain, which is one of the main components of contractile muscle fibers (myofibrils), and disruption of their functioning can lead to various muscle diseases [40,41]. The *TTN* (Titin) gene (log2FoldChange = −12) encodes the titin protein, which helps maintain the elasticity and stability of sarcomeres—the functional units of muscle fibers. When this protein is disrupted, tibial muscular dystrophy is observed, which is characterized by progressive muscle weakness, especially in the shin area [42]. The *MYOT* gene (log2FoldChange = −12) encodes a protein that is a key component of the Z-disk and directly binds to F-actin. It is known that heterozygous mutations in this gene can lead to late-onset distal myopathy [43].

Studies of the cellular and molecular processes associated with BRKS are limited and should be developed, as the heterogeneity of symptoms and the diversity of disease phenotypes suggest that a large number of factors—genetic, epigenetic, and environmental—are involved in its initiation and progression. The use of cellular models will enable the assessment of mutation-induced impairments, but studies such as transcriptome analysis will help in understanding which cellular processes are altered and what needs further attention.

## 3. Materials and Methods

### 3.1. Plasmids

Total RNA was extracted from the HeLa cell line (Cell Line Collection, National Research Center, Kurchatov Institute—PNPI) using the ExtractRNA reagent (Evrogen, Russia) according to the manufacturer’s instructions. C-DNA was synthesized with the help of the MMLV RT kit (Evrogen, Moscow, Russia) and 094 primer (Table 2).

Wild-type *PLOD2* (*LH2a*) was amplified from cDNA with 110 and 111 primers of Table 2. Primer 110 (Figure S2) included the Kozak consensus and BsaI recognition site (GGTCTC) prior to the start codon. The PCR fragment was then purified from the reaction mixture using the CleanupMini kit (Evrogen, Moscow, Russia). A PCR fragment of 2261 bp was treated with the BsaI-HF restriction enzyme, and the resulting DNA fragment was extracted from a 0.9% agarose gel using the Cleanup Standard kit (Evrogen, Moscow, Russia). After BsaI endonuclease cleavage, EcoRI sticky ends were generated. The PCR fragment was purified from the reaction mixture using the CleanupMini kit.

The wild-type *PLOD2* (*LH2b*) was made by site-directed mutagenesis. In the first stage, two DNA fragments (1.5 and 0.8 kb) were amplified using cDNA as a template and 095/122 and 121/094 primers of Table 2 accordingly. The 121 and 122 primers (Figure S3) included 13A exon and BsaI recognition sites (GGTCTC). BsaI-digested PCR fragments were purified by the CleanupMini kit and ligated using T4 DNA Ligase (Evrogen, Moscow, Russia). Then, 1 µL of the ligation mixture was a template for PCR with 110 and 111 primers of Table 2. A 2.3 kb PCR fragment was extracted from 0.9% agarose gel using a commercial Cleanup Standard kit followed by BsaI-HF treatment (New England BioLabs, Ipswich, MA, USA). The DNA fragment was then purified from the reaction mixture using the CleanupMini kit.

The mutation Thr629Ala (A1885G) was introduced by site-directed mutagenesis. In the first stage, two DNA fragments, 1.5 and 0.8 kb, were amplified using *PLOD2* (*LH2b*) as a template and 095/091 and 090/094 primers of Table 2 accordingly. The 090 and 091 primers (Figure S4) included BsaI recognition sites (GGTCTC). BsaI-digested PCR fragments were purified by the CleanupMini kit and ligated using T4 DNA Ligase. Then, 1 µL of the ligation mixture was a template for PCR with 110 and 111 primers (Table 2). A 2.3 kb PCR fragment was extracted from a 0.9% agarose gel using the commercial Cleanup Standard kit, followed by BsaI-HF treatment. The DNA fragment was then purified from the reaction mixture using the CleanupMini kit.

The *GFP* was amplified using the pAAV-GFP vector (Cellbiolabs, San Diego, CA, USA) as a template and 112 (Figure S5) and 113 primers of Table 2. The PCR product was then purified by the CleanupMini kit and digested with BsaI-HF and HindIII-HF restriction endonucleases (New England BioLabs, Ipswich, MA, USA). Then, the resulting 0.9 kb PCR fragment was purified by the CleanupMini kit.

The pTCN plasmid (NovoPro Bioscience Inc., Shanghai, China) was digested by EcoRI-HF and HindIII-HF enzymes, followed by incubation with rSAP (Shrimp Alkaline Phosphatase, New England Biolabs). The resulting 5.7 kb DNA fragment was extracted from a 0.9% agarose gel and purified using a commercial Cleanup Standard kit.

The DNA concentrations were determined using a NanoPhotometer P-Class P-300 (Implen, München, Germany) for subsequent ligation reaction using T4 DNA ligase. The cloning scheme is presented in Table 3.

The molar ratio of insert to vector in the ligation mixture was (5–10):1. Ligation was carried out overnight at 12 °C. The transformation of C2523 competent cells (New England BioLabs) by ligation mixtures was performed using the standard electroporation method. Then, the cell suspension was plated on LB agar medium supplemented with the antibiotic ampicillin (100 µg/mL). The plates were incubated at 37 °C for 24 h. Ten colonies with typical morphometric characteristics were selected and inoculated into separate sterile 10 mL tubes containing 2.5 mL of liquid LB medium with ampicillin (100 µg/mL). The *E. coli* cultures were cultivated for 16 h in a shaker incubator (Inforce, Bottmingen, Switzerland) at 37 °C and 180 rpm. An amount of 500 µL of the culture was transferred to cryovials containing 500 µL of sterile 50% glycerol and stored in a freezer at −80 °C. DNA was extracted from a 2 mL culture using the Plasmid Miniprep kit (Evrogen, Moscow, Russia).

The primers were designed by Vector NTI software (version 8) and synthesized by Evrogen (Moscow, Russia).

### 3.2. Clone Selection by PCR, Restriction Analysis, and Sequencing

The initial analysis of the authenticity of the constructs was performed by PCR. The 114 and 124 primers were used to verify the genetic constructs. The PCR products were loaded onto a 1% agarose gel in TAE buffer. A total of 3.2 kb of PLOD2-GFP genes were sequenced on a “Nanofor 05” genetic analyzer (Syntol, Moscow, Russia) at the National Research Center, Kurchatov Institute—PIKF, and analyzed by using “Chromas Lite, version 2.1.1” and “Vector NTI Suite 8” software. Clones with correct sequences were selected for further investigations.

### 3.3. Cell Cultures

DF2 cells, human dermal fibroblasts (the shared research facility “Vertebrate Cell Culture Collection”, Saint-Petersburg, Russia), and HEK293 cells (Human Embryonic Kidney; collection at the Petersburg Nuclear Physics Institute, named by B.P. Konstantinov of the National Research Center, Kurchatov Institute, Gatchina, Russia) were cultured on DMEM F12 medium supplemented with glutamine (Biolot, Saint Petersburg, Russia), antibiotics (penicillin and streptomycin (Biolot, Saint Petersburg, Russia)), and 10% FBS (Biolot, Saint Petersburg, Russia) under standard conditions (37 °C, 5% CO_2_).

### 3.4. Transfection

Cells were grown on plates (Jet Biofil, Guangzhou, China) or 35 mm Petri dishes (Jet Biofil, Guangzhou, China) during the day. Once a monolayer confluence of approximately 60–70% was achieved, the HEK293 cells were transfected using GenJect-39 (MOLECTA, Moscow, Russia) following the manufacturer’s instructions. After 16 h of incubation, the transfection medium was removed. Then, cells were maintained in medium containing 1.5 mg/mL of neomycin (HiMedia, Maharashtra, India).

Once DF2 cells had grown to 80% confluence, the cells were transfected using GenJect-39 (MOLECTA, Moscow, Russia). After 6 h of incubation, the transfection medium was removed, and cells were incubated in complete medium, as described in Section 3.3 Transfection efficiency was assessed using CytoFLEX B3-R2-V2 flow cytometer (Beckman Coulter, Brea, CA, USA and EVOS™ FL Auto Imaging System (Thermo Fisher Scientific, Waltham, MA, USA) to detect GFP-positive cells.

### 3.5. Flow Cytometry

A period of 24 h after transfection, the cells were washed with Versene solution (Biolot, St. Petersburg, Russia), disaggregated with trypsin–versene solution (1:1; Biolot, St. Petersburg, Russia), and centrifuged for 5 min at 1000 rpm, and then the supernatant was removed. The cells were resuspended in 300 µL of medium and analyzed on a CytoFLEX B3-R2-V2 flow cytometer (Beckman Coulter, Brea, CA, USA; 20,000 cells per experiment).

### 3.6. Analysis of Localization of PLOD2

HEK293 cells and DF2 cells transfected with pTCN were grown on glass-bottom cell culture dishes. After 24 h, the culture medium was removed, and 100 μL of ER staining solution was applied to the glass bottom for 30 min, according to the manufacturer’s protocol (CytoPainter ER Staining Kit, ab139482, Abcam, Cambridge, UK). After being washed twice with 1X Assay Buffer, cells were immediately imaged on a confocal microscope without removing the Assay Buffer. HEK293 cells were analyzed using a Leica TCS SP5 confocal microscope (Leica, Wetzlar, Germany). DF2 cells were immediately imaged on an EVOS™ FL Auto Imaging System (Thermo Fisher Scientific, Waltham, MA, USA). DF2 cells were additionally stained with antibodies to the PLOD2 protein (1:50, PAE257Hu01, Cloud-Clone Corp., Katy, TX, USA) and Alexa-Fluor594 (1:400, ab150080, Abcam, Cambridge, UK) to confirm transfection.

### 3.7. Analysis of Cellular Effects of PLOD2

#### 3.7.1. Analysis of Cell Viability by the MTT Assay

HEK293 cells in each group were diluted to a certain concentration, seeded in a 96-well plate (approximately 5 × 10^3^ cells per well), and incubated (CO_2_ 37 °C) for 24, 48, or 120 h after transfection. Then, 10 µL of MTT solution was added to each well for 3 h and incubated in the dark. After that, the culture medium in each well of the plate was carefully removed, followed by the addition of 100 µL of DMSO, and the plate was shaken (30 min) to dissolve. DF2 cells in each group were diluted, seeded in a 24-well plate, and incubated (CO_2_ 37 °C) for 24, 48, and 120 h after transfection. After that, the culture medium in each well was replaced with 300 μL of medium containing 30 μL of MTT reagent for 3 h. The culture medium was then removed, and 300 µL of DMSO was added to each well and kept in the dark for 30 min. An amount of 100 µL was dispensed into each well of a 96-well plate.

The absorbance of each well was measured using a Multiskan FC spectrophotometer (Thermo Fisher Scientific, Waltham, MA, USA) at a wavelength of 540 nm. The obtained data were processed using the KyPlot 6.0 program (KyensLab Inc., Tokyo, Japan), and the Tukey–Kramer test was conducted. Statistical significance was considered at *p* < 0.05.

#### 3.7.2. Matrigel Adhesion Assay

The adhesion rate of HEK293 cells was analyzed 24 h after transfection, as described in [44]. Briefly, cells were added to a 96-well plate coated with 100 μL of ABW Matrix (Shanghai Nova Pharmaceutical Technology Co., Ltd., Shanghai, China) and incubated at room temperature. After an hour, 1 × 10^4^ cells were placed into each well. After 30/60/90 min, the non-adherent cells were removed, and the attached cells were photographed using an EVOS™ FL Auto Imaging System (Thermo Fisher Scientific). Images were analyzed using ImageJ (version 1.53a). Four replicates were performed per point in each experiment. The experiments were repeated 3 times.

#### 3.7.3. Scratch Migration Assay

The scratch migration assay was performed using a 35 mm µ-Dish with 2-well culture inserts (Ibidi, Gräfelfing, Germany), following the manufacturer’s instructions. The images were captured after 0, 2, 24, and 48 h for HEK293 cells and 0, 2, 24, 48, 72 and 120 h for DF2 cells. The experiment was carried out in 4 replicates.

### 3.8. Transcriptomic Analysis

#### 3.8.1. Muscle Samples

This research study was approved by the Local Ethics Committee of the H. Turner National Medical Research Center for Children’s Orthopedics and Trauma Surgery of the Ministry of Health of the Russian Federation, No. 19-3 of 9 December 2019. The analysis was performed on a muscle biopsy (quadriceps femoris) from an 8-year-old female patient diagnosed with BRKS. A muscle sample was collected during elective corrective surgical procedures and was snap-frozen in liquid nitrogen and stored at −80 °C until processing. Skeletal muscle transcriptome sequencing samples from the open-source GEO (accession number GSE201255) were used as controls [38].

#### 3.8.2. Preparation of Libraries

To isolate total cellular RNA, fragments of muscle tissue were washed in phosphate-buffered saline by centrifugation at 1200× *g* for 4 min at 4 °C, and this procedure was repeated three times. Subsequently, the tissue was homogenized by repeated freezing in liquid nitrogen, followed by grinding with a pestle. Total RNA was extracted using the TRIzol reagent. To purify total RNA from genomic DNA, 1500 ng of the total RNA solution obtained was transferred to a separate tube and treated with DNase I according to the standard protocol.

To deplete ribosomal RNA, the Library Preparation VAHTS mRNA Capture Beads kit was utilized in accordance with the standard protocol. The efficacy of rRNA purification was subsequently assessed via real-time PCR. The concentration of the resultant total RNA was quantified using a fluorometer (Qubit 4 Fluorometer, Thermo Fisher Scientific, Waltham, MA, USA) with a Qubit RNA HS Kit, adhering to the manufacturer’s standard protocol. The quality of RNA and the degree of purification were evaluated using a NanoDrop OneC instrument (Thermo Fisher Scientific, Waltham, MA, USA) by examining the A260/A280 and A260/A230 wavelength ratios.

RNA libraries were prepared utilizing the MGIEasy RNA Directional Library Prep Set (MGI, Shenzhen, China), in accordance with the manufacturer’s protocol. Sequencing was conducted on the DNB-SEQ-G400 platform (MGI, Shenzhen, China) in paired-end reading mode, with a read length of 100 bp.

#### 3.8.3. RNA-Sequencing Data Processing

The quality of the obtained data was assessed utilizing FastQC software (version 0.12.0). When necessary, adapter trimming and filtering of low-quality reads were conducted using Trimmomatic (version 0.33) [45]. Read mapping to the reference genome (GRCh38/hg38) was performed using Hisat2 software (version 2.2.1) [46]. The htseq-count program (version 2.2.1) was employed to enumerate reads for each transcript [47]. Differential expression analysis was conducted utilizing the DESeq2 package (version 1.46.0) in R (version 4.2.0) [48]. Genes exhibiting a *p*-value less than 0.01 in the analysis were classified as differentially expressed genes. DEGs with a log2FoldChange (log2FC) threshold exceeding 4 were categorized as upregulated, whereas those with log2FC below −4 were categorized as downregulated.

#### 3.8.4. Functional and Enrichment Analysis of DEG Pathways

Gene Ontology (GO) pathway enrichment analyses were conducted to elucidate biological processes, cellular components, and molecular functions. The Bioconductor package ‘org.Hs.eg.db’ (version 3.20.0) and the ‘clusterProfiler’ package (version 4.14.4) [49] were used for GO pathway enrichment analyses in the categories of biological processes (BPs), molecular functions (MFs), and cellular components (CCs). For the enrichment of human phenotypes (Monarch), the online resource STRING (version 12.0) was used [50].

### 3.9. Evolutionary Comparison of Protein Sequences

The amino acid sequences of proteins were retrieved from the NCBI database and aligned using the MEGA program (version 11.0).

## Figures and Tables

**Table 1:** Amino acid sequence of human lysyl hydroxylase LH2 aligned with amino acid sequences of other human lysyl hydroxylases (LH1 and LH3) and lysyl hydroxylases from different organisms. Amino acid substitution is highlighted in black, while homologous lysyl hydroxylase sequences are highlighted in gray.

*Homo sapiens* LH2	G	G	Y	E	N	V	P	T	D	D	I	H	M
*Homo sapiens* LH1	G	G	Y	E	N	V	P	T	I	D	I	H	M
*Homo sapiens* LH3	G	G	Y	E	N	V	P	T	V	D	I	H	M
*Mus musculus* LH1	G	G	Y	E	N	V	P	T	I	D	I	H	M
*Mus musculus* LH2	G	G	Y	E	N	V	P	T	D	D	I	H	M
*Mus musculus* LH3	G	G	Y	E	N	V	P	T	V	D	I	H	M
*Rattus norvegicus* LH1	G	G	Y	E	N	V	P	T	I	D	I	H	M
*Rattus norvegicus* LH2	G	G	Y	E	N	V	P	T	D	D	I	H	M
*Rattus norvegicus* LH3	G	G	Y	E	N	V	P	T	V	D	I	H	M
*Danio rerio* LH1	G	G	Y	E	N	V	P	T	I	D	I	H	M
*Danio rerio* LH2	G	G	Y	E	S	V	P	T	D	D	I	H	M
*Danio rerio* LH3	G	G	Y	E	N	V	P	T	V	D	I	H	M
*Drosophila melanogaster* LH	G	G	Y	E	A	V	P	T	R	D	I	H	M
*Caenorhabditis elegans* LH	G	G	Y	E	N	V	P	T	R	D	I	H	M

**Table 2:** List of primers used in this study.

Primer	The Nucleotide Sequence 5′→3′	Restriction Site	Application
083	CTTCCATCAAGCTTAGGGATCTATAAATGACACTGCAATG	HindIII	*PLOD2* amplification
090	TAACAACGGTCTCATCCACATGAAGCAAGTTGATCTG	BsaI	
094	CTTAGGGATCTATAAATGACACTGC	-	
095	AATATGGGGGGATGCACGGT	-	
110	TAACAACGGTCTCTAATTGCCACCATGGGGGGATGCACGGTG	BsaI	
121	TAACAACGGTCTCAGAAACATTCCAAATGCTCAGCCCCCCAAAGGGTGTATTTATGTACATTTCTAATAGAC	BsaI	13A exon amplification
122	CTTCCATCGGTCTCATTTCCGGAGTAGGGGAGTCTTTTTCCCTTTGTAAAGTCATTTCTCTAGCATTTCGGC	BsaI	
111	ATTCAAGGTCTCACCATTCCACCACCGGGATCTATAAATGACACTGCAATG	BsaI	*PLOD2* amplification (without stop-codon)
091	CTTCCATCGGTCTCTTGGATATCATCAGCTGGGACA	BsaI	Thr629Ala mutation
112	TTGGAGGTCTCTATGGTGAGCAAGGGCGAGG	BsaI	*GFP* amplification
113	AGAAGGACACCTAGTCAGAC	-	
084	TGTGAAGGTCCTTGGTCAAG	-	Confirmation of the plasmids
085	CCAGACCTTAAATGGAGCTG	-	
086	CATGGGAATGGACTTTTGCC	-	
087	CCTACTCCGGAAACATTCCA	-	
103	TTCCCAATGTGCACAACAGG	-	
104	AGAAAAGGGGTTGGTTGCTC	-	
105	ATGTTTCCGGAGTAGGGGAG	-	
114	CTGGATTATTCTGAGTCCAAGC	-	
124	CTGCTATTGTCTTCTAGAAGGC	-	

Green—restriction sites; orange—mutations; blue—Kozak sequence.

**Table 3:** Cloning scheme.

Genetic Construct	Cloning Scheme
LH2a WT + GFP	pTCN, EcoRI-HF/HindIII-HF—5670 kb
	PCR1(cDNA, 110/111—2261 bp), BsaI-HF
	PCR2 (pAAV-GFP/NcoI, 112/113—937 bp), BsaI-HF/HindIII-HF
LH2b WT + GFP	pTCN, EcoRI-HF/HindIII-HF—5670 kb
	PCR1(cDNA, 095/122—1555 bp), BsaI-HF PCR2(cDNA, 121/094—759 bp), BsaI-HF PCR3[lig(PCR1/BsaI-HF, PCR2/BsaI-HF) 110/111—2300 bp], BsaI-HF
	PCR4 (pAAV-GFP/NcoI, 112/113—937 bp), BsaI-HF/HindIII-HF
PLOD2 Thr629Ala (LH2b) + GFP	pTCN, EcoRI-HF/HindIII-HF—5670 kb
	PCR1(LH2b WT + GFP, 095/091—1990 bp), BsaI-HF PCR2(LH2b WT + GFP, 090/094—401 bp), BsaI-HF PCR3[lig(PCR1/BsaI-HF, PCR2/BsaI-HF) 110/111—2300 bp], BsaI-HF
	PCR4 (pAAV-GFP/NcoI, 112/113—937 bp), BsaI-HF/HindIII-HF

## Data Availability

The data presented in this study are available in the article and Supplementary Materials.

## Supplementary Materials

The following supporting information can be downloaded at: https://www.mdpi.com/article/10.3390/ijms252413379/s1.
