# Supplementary material for: Cellular and Molecular Effects of the Bruck Syndrome-Associated Mutation in the PLOD2 Gene
Source: Int J Mol Sci. 2024 Dec 13;25(24):13379. doi: 10.3390/ijms252413379 (PMC11676324; doi:10.3390/ijms252413379)
Supplement: Supplementary file 1 [file ijms-25-13379-s001.zip › Supplementary material Figure S1.pdf]

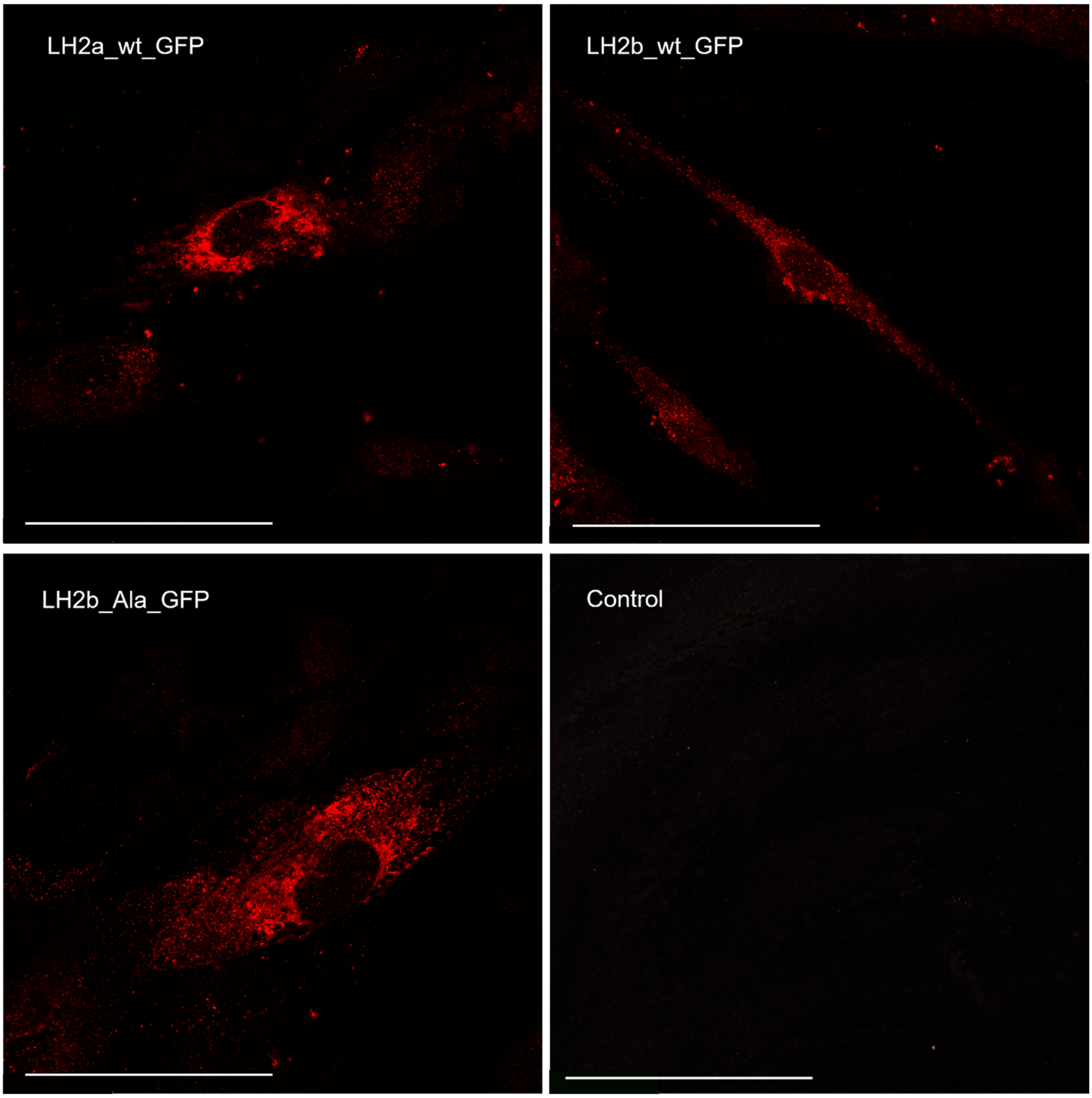

Figure S1. Localization of PLOD2 protein in DF2 cells transfected with three different constructs, including GFP-LH2a, GFP-LH2b, and GFP-LH2b with mutation Thr629Ala and Control without constructs. Red – PLOD2 protein. Scale bar – 100 µm.
