# Supplementary figures and images for: Cellular and Molecular Effects of the Bruck Syndrome-Associated Mutation in the PLOD2 Gene
Source: Int J Mol Sci. 2024 Dec 13;25(24):13379. doi: 10.3390/ijms252413379 (PMC11676324; doi:10.3390/ijms252413379)

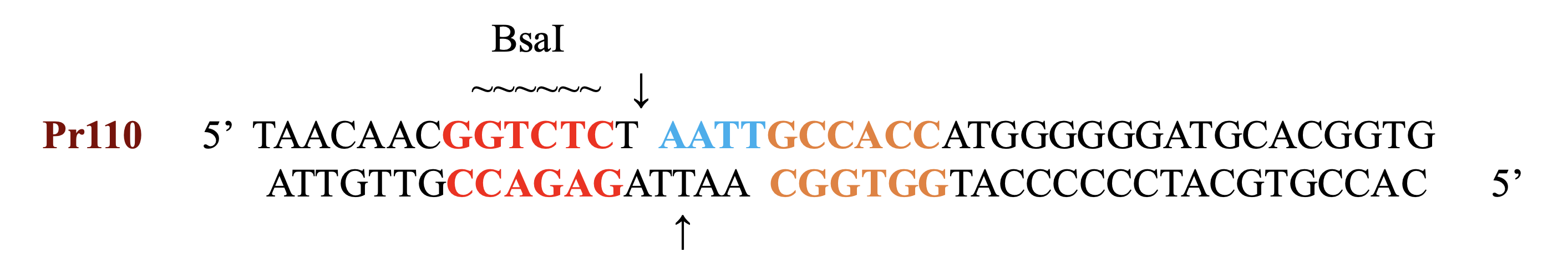

Supplement: Supplementary file 1 [file ijms-25-13379-s001.zip › Supplementary material Figure S2.png]

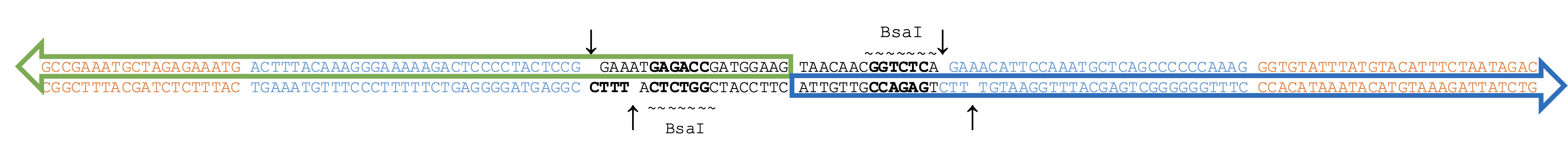

Supplement: Supplementary file 1 [file ijms-25-13379-s001.zip › Supplementary material Figure S3.png]

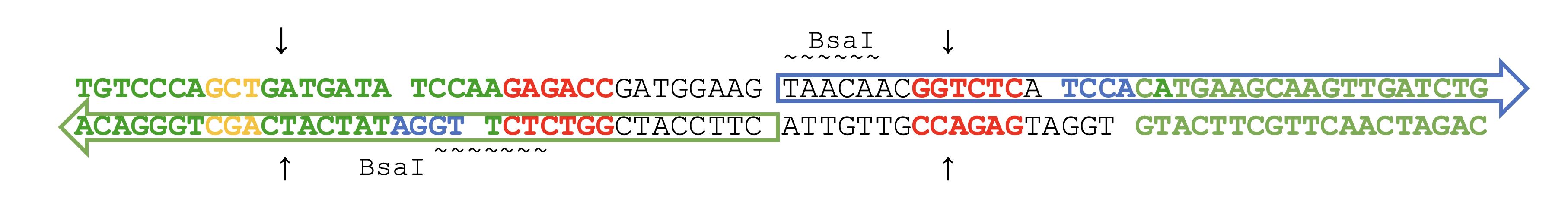

Supplement: Supplementary file 1 [file ijms-25-13379-s001.zip › Supplementary material Figure S4.png]

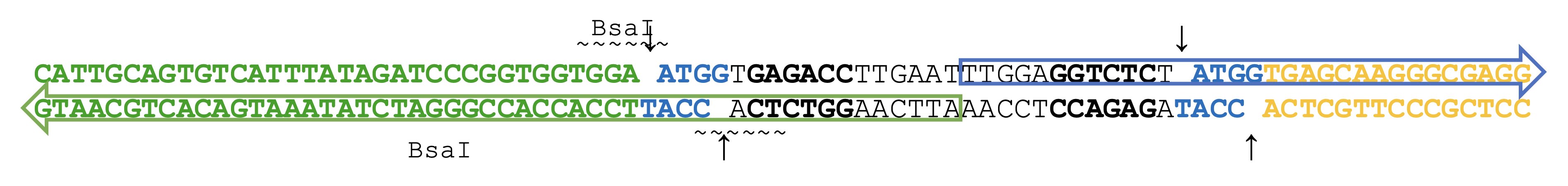

Supplement: Supplementary file 1 [file ijms-25-13379-s001.zip › Supplementary material Figure S5.png]
